# Serum Level of Interleukin-6 in Patients with Oral Tongue Squamous cell Carcinoma

**Published:** 2015-05

**Authors:** Alireza Lotfi, Nikzad Shahidi, Gholamreza Bayazian, Shahin AbdollahiFakhim, Rasoul Estakhri, Ali Esfahani, Rezvan Notash

**Affiliations:** 1 *Hematology and Oncology Research Center, Tabriz University of Medical Sciences, Tabriz, Iran.*; 2 *Department of Otorhinolaryngology, Imam Reza Hospital, Faculty of Medicine, Tabriz University of Medical Sciences, Tabriz, Iran.*; 3 *Department of Otorhinolaryngology,Hazrat Rasool hospital, Faculty of Medicine, Iran University of Medical Sciences, Tehran, Iran.*; 4 *Department of Pathology, Imam Reza hospital, Faculty of Medicine, Tabriz University of Medical Sciences, Tabriz, Iran.*

**Keywords:** Cytokines, Interleukin-6, Oral tongue carcinoma, Squamous cell carcinoma

## Abstract

**Introduction:**

The clinical outcome of patients with squamous cell carcinoma (SCC) located in the head and neck has remained poor despite ongoing advances in diagnosis and management. Interleukin-6(IL-6) is a multi-functional cytokine that plays an important role in the process of cell differentiation and is increased in several malignancies. The aim of this study was to investigate the serum levels of interleukin-6 in patients with oral tongue SCC.

**Materials and Methods:**

In a cross-sectional study, 17 patients with oral tongue SCC were compared with the same number of age- and gender-matched healthy subjects. Serum IL-6 level fluctuation was determined using an immunological technique, before detecting its possible association with the subjects’ age, gender, drinking and smoking history, cancer site, and disease severity.

**Results:**

The intensity of serum IL-6 in patients with oral tongue SCC was statistically significantly higher than that in healthy subjects (P<0.001). Serum IL-6 level was independent of the patients’ age, gender, smoking and drinking history as well as cancer stage.

**Conclusion:**

IL-6 is a valuable biomarker in the diagnosis of oral tongue SCC. Its high sensitivity makes prediction of this condition possible, while this biomarker can also be used to screen high-risk patients.

## Introduction

Cancer of the oral cavity is the sixth most frequently observed type of cancer in the world, and more than 90% of reported oral cancers are squamous cell carcinomas (SCC) (1). Oral tongue SCC (OTSCC) is the most common cancer diagnosed in the oral cavity, accounting for 20–40% of oral carcinomas (2). Despite advances in surgery, radiation and chemotherapy, the oral cancer 5-year survival rate has not significantly improved over recent years, and remains at approximately 50–55% (1). In fact, despite improvements in locoregional control, morbidity and mortality rates in these cancers have not significantly improved over the past three decades (1,3). 

The mortality rate in oral SCC can be effectively controlled through efforts towards early diagnosis and prevention (3). SCC of the head and neck is usually diagnosed in the absence of specific symptoms, which in many cases may delay diagnosis. Therefore, availability of sensitive and specific biomarkers to achieve early diagnosis would be highly beneficial (4).

Interleukin-6 (IL-6) is a multi-functional cytokine that plays an important role in differentiation and growth factors for a variety of cells such as B-cells, T-cells, neuronal cells, osteoclasts, and endothelial cells (5). Several human tumor cells, including esophageal SCC, multiple myeloma and lung carcinoma, have been reported to produce IL-6 (6). Thus, the aim of this study was to evaluate the secretion level of IL-6 in OTSCC and to investigate its possible association with severity and risk factors.

## Materials and Methods

Seventeen patients with OTSCC in the North-West of Iran referred to the Otolaryngology Clinic at Imam Reza Hospital (Tabriz University of Medical Science) were enrolled in this study. Seventeen healthy age- and gender-matched individuals were selected as a control group. Exclusion criteria included a history of recent traumas, acute infections, recent burns, lacerations, previous surgery, chemotherapy or radiotherapy. All patients were recently diagnosed with a primary OTSCC. Control variables were age, sex, smoking history, the cancer site and stage. In the control group, no subjects had a record of recent trauma, laceration, infection, illness, previous surgery, smoking or drinking problems. Control subjects underwent a physical examination before selection. 

A 3-ml blood sample was taken from every subject prior to surgery. The centrifuged blood samples were stored at −70 °C before testing. The intensity of serum IL-6 was determined by using a quantitative sandwich enzyme-linked immunosorbent assay (ELISA) technique (eBioscience- Platinum ELISA-MedSystems Gmbh, Vienna, Austria). The data were tabulated using SPSS 17 (Polar Engineering and Consulting) for statistical analysis. P<0.05 was set as the critical level of significance. Descriptive data are presented as percentiles, while a Chi-square test was conducted to analyze qualitative data. The qualitative data obtained from the small number of subjects were analyzed after running a non-parametric Mann Whitney U-test. To estimate the correlation index between IL-6 range and age, a Kendall test of correlation was performed, while the sensitivity level of IL-6 was tested using receiver operating characteristic (ROC). Similarly, P<0.05 was set as the critical level of significance. 

## Results

**Fig 1:**
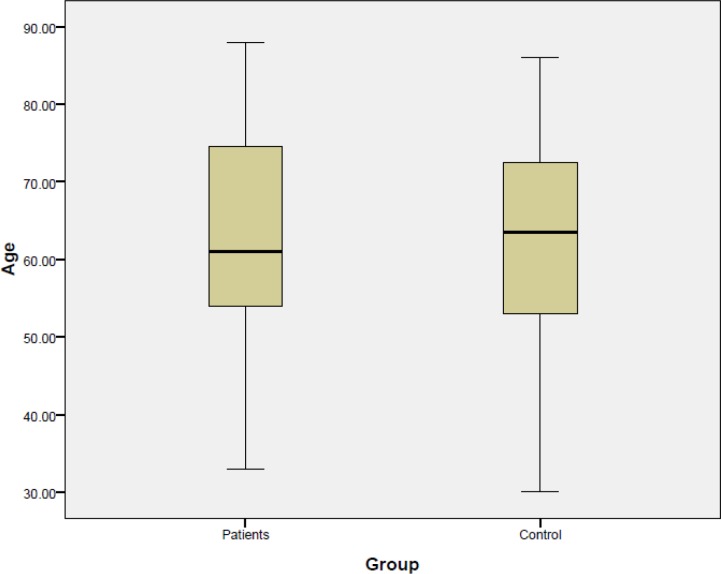
Average age in the treatment and control groups

Seventeen patients were enrolled with OTSCC and 17 age- and gender-matched healthy controls were enrolled in this study. There were eight males and nine females in both groups. The mean age of the patients was 64.2±14.02 years, compared with 62. 17±14.23 years in the control group(Fig.1).

**Fig 2:**
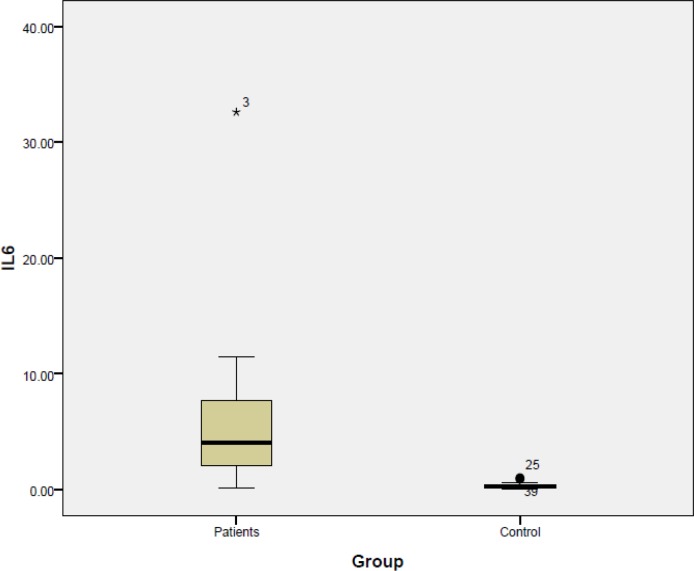
Intensity of serum IL-6 levels in treatment and control groups

The average serum IL-6 level in patients with oral cavity SCC was 5.8 pg/ml compared with the much lower value of 0.2 pg/ml in the control group (P<0.001) (Fig.2).

One patient had a smoking history, involving use of around 25±12.9 packs per year. The average serum IL-6 level among non-smokers was 11.5±0.0 pg/ml compared with 6.13±7.16 pg/ml in this patient (P<0.05). 

Stage of cancer progression at the time of the study did not seem to affect intensity of serum IL-6 levels (8.1±1.3pg/ml, 4.21±2.85 pg/ml, and 7.25±3.35 pg/ml for Stage 1 (n=3), Stage 2 (n=8) and Stage 3 (n=6), respectively; P<0.67). Similarly, elevated serum IL-6 level was not associated with patients’ gender (P<0.58), age (P<0.08) or positive/negative lymph nodes (P<0.757). As none of the patients had a history of drinking alcoholic beverages, any possible connection between this risk factor and serum IL-6 level was undetectable. Statistical details are summarized in Table 1.

**Table 1:** Research findings

**Variable**	**Mean serum ** **IL-6 (pg/ml)**	**P-value**
**Sex**		
Male	7.5±10.65	0.58
Female	5.47±3.2	
TNM Stage		
Stage 1	8.1±1.3	0.67
Stage 2	4.21±2.85	
Stage 3	7.25±3.35	
Lymph node involvement		
Positive	6.01±9.1	0.757
Negative	7.25±3.35	
Addiction		
Smoker	6.13±7.16	0.5
Non-smoker	11.5±0.0	

**Fig 3:**
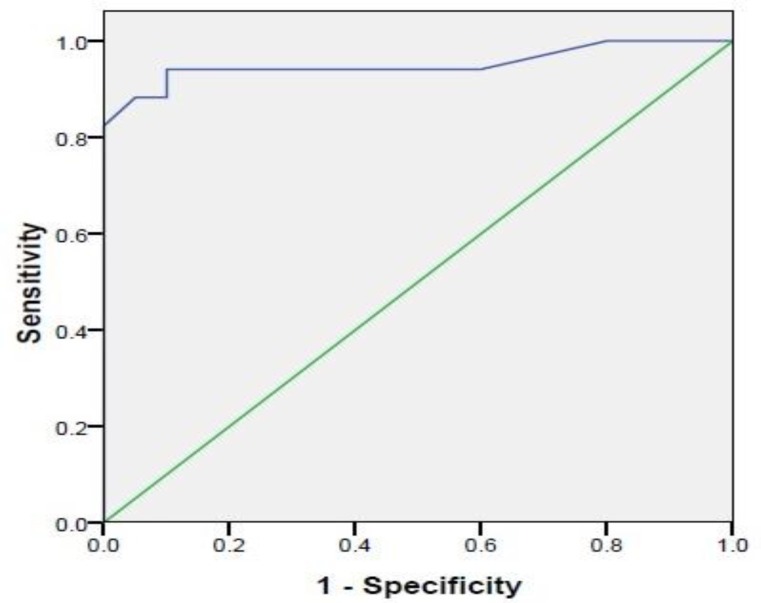
Sensitivity of IL-6 in oral tongue squamous cell carcinoma patients

In patients affected with OTSCC, the serum IL-6 sensitivity was high, at close to 95% (Fig.3).

## Discussion

Head and neck SCC is reported to threaten the lives of 50,000 people in the United States each year; 13,000 of whom do not survive (7). Advances in surgical and non-surgical techniques have been successful in controlling this cancer, although they have not affected rates of mortality or relapse. Recently, the development of histological markers and inflammatory mediators that enable us to detect the potential for progression and aggressive growth have become very popular (7,6). Numerous studies support the fact that multiple biomarkers are released in head and neck carcinomas (8). Inflammatory conditions in the oral cavity increase cytokine production, leading to raised serum levels of IL-1, 6, 8 and tumor necrosis factor-alpha (TNF-α). Increased serum levels of these markers provides the potential for their use in the prediction and treatment of head and neck SCC (9,10).

In particular, IL-6 and IL-8 are important factors in predicting cell differentiation and growth of tumor cells. Their measurable increase in renal cell carcinoma, OSCC and lymphoma has been reported (6,11). 

In one study, salivary levels of IL-1α, IL-6, IL-8, vascular endothelial growth factor A (VEGF-a) and TNF-α were able to predict the progression of tongue SCC from high-risk to neoplasm, serving as potential biomarkers for cancer screening and early detection (12).

In a study by Rhodus et al., an elevated saliva intensity of TNF-a, and IL-1, 6, 8, in oral cavity SCC was detected. In this study, it was shown that in moderate and severe Lichen planus dysplasia, the saliva intensity of TNF-α, IL-1, 6, 8 was much higher than that in healthy individuals (13). The authors argue that while the intensity of TNF-α and IL-1 increased to levels similar to those detected in oral SCC, the levels of IL-6 and IL-8 remained lower in intensity than those seen in oral SCC. This finding indicates that IL-6 and IL-8 may be remarkable factors in oral cavity SCC. This finding was supported in a study by Saheb Jame et al. who examined a very high saliva intensity of IL-6 up to 15.9 pg/ml in oral SCC (14). This is contrary to the slow and insignificant secretion of IL1, 8 and TNF-α in the patients’ blood. 

The current study greatly supports the increase of IL-6 in oral cavity SCC. Moreover, the serum level of IL-6 showed a high diagnostic sensitivity of nearly 95% in patients with oral cavity SCC.

This finding was more in line with those of Duffy et al. and Hamad et al. who measured a sensitivity of 80% in the patients’ serum and 73% in their saliva. They acknowledged IL-6 as a determining biomarker in oral cavity SCC (15). Unlike Saheb Jame et al., Hamad et al. reported a higher increase of serum IL-8 sensitivity, close to 90%, in oral cavity SCC (16). 

Despite the controversies, IL-6 is suggested as a biomarker for early diagnosis and follow-up to detect relapse in oral cavity carcinomas (16). Since patients are commonly diagnosed with oral cavity carcinoma at the later stages of the disease, it is expected that serum IL-6 levels increase with cancer stage. The increasing inflammation and lesion region would increase the serum IL-6 level.

Masaaki et al. reported the remarkably high serum IL-6 levels in invasive tumors (17). In their study, a strong connection existed between serum IL-6 levels and excessive weight loss, invasiveness of the tumor to surrounding tissues, lymph node involvement, and impossibility of complete tumor resection. Similar findings were reported by Riedel et al. between the average serum IL-6 level and the cancer stage (6). In other cases with lymph node involvement, a higher serum IL-6 was also observed. 

Contrary to the previous studies, the current researchers could not detect any association between serum IL-6 levels and cancer stage in OTSCC. A probable reason was the small number of patients in this study that precluded any general statements. Moreover, the researchers in this study found no connections between their patients’ gender or age and serum IL-6 sensitivity levels. This reinforces the reliability of serum IL-6 as an independent biomarker in patients diagnosed with OTSCC. Similar findings by St. John et al. supported the lack of connection between gender, age or drinking and smoking history in the patients and their serum IL-6 levels. In conclusion, among biomarkers such as saliva level, serum IL-6 has a higher diagnostic significance in OCSCC. 

## Conclusion

Interleukin-6 is a valuable biomarker for the diagnosis of head and neck SCCs. It has a high sensitivity in predicting these patients and is recommended for use in screening high-risk cases. 
